# Minimally invasive percutaneous cannulated polyaxial screw and rod fixation thoracolumbar and lumbosacral vertebrae: cadaveric evaluation of accuracy and safety of a fluoroscopic-guided technique, in large breed dogs

**DOI:** 10.3389/fvets.2026.1789511

**Published:** 2026-03-20

**Authors:** Francisca Couto, Jeremy Rose, Victor Nores, Colin J. Driver

**Affiliations:** 1 Lumbry Park Veterinary Specialists, Alton, United Kingdom; 2 AURA Veterinary, Guilford, United Kingdom

**Keywords:** fluoroscopy, implant accuracy, vertebral column, vertebral fixation, veterinary spinal surgery

## Abstract

**Objective:**

To develop and report the accuracy and safety of a technique for percutaneous pedicle screw-rod fixation of the thoracolumbar and lumbosacral regions using fluoroscopic guidance in dog cadavers, comparing two levels of surgical experience.

**Study design:**

Cadaveric methods comparative study.

**Animals:**

Cadaveric vertebral columns of dogs (*n* = 3).

**Methods:**

Pre-operative computed tomography (CT) scans facilitated in-silico surgical planning. Ideal implant insertion points and trajectory were recorded, and the image was subsequently reconstructed to mimic the appearance of intra-operative fluoroscopy. After surgical preparation from T8 to S2, the study was divided in two parts: part one to evaluate safety and accuracy of percutaneous “end-on” fluoroscopic drill hole position technique (EOFG); and part two to determinate the feasibility of thoracolumbar (T13-L1) and lumbosacral (L7-S1) percutaneous cannulated pedicle screw and rod fixation (PC-PSRF). The procedures were performed by one of two experienced ECVN diplomates, and one ECVN neurology resident (inexperienced in spinal fixation surgery). Following post-operative CT imaging, drill-hole safety was established using a modified Zdichavsky classification and optimal placement was compared between surgeons. Drill-hole accuracy was calculated based on the deviation from planned angle and bone purchase, between the two levels of surgeon experience. Data sets were analysed at both univariable and multivariable levels with logistic regression analysis.

**Results:**

No unsafe holes (0/34) were drilled by experienced surgeons, whereas the inexperienced surgeon drilled 4/34 (11.8%) unsafe holes. Optimal drill-hole placement was significantly associated with surgeon experience (*p* < 0.001, OR = 9.66), whereas cadaver number and spinal region did not reach significance. Drill-hole angle and bone depth deviations were significantly different between experienced and inexperienced surgeons (*p* = 0.04 and *p* < 0.001, respectively). Angle deviations were not significantly different between experience levels by region, whereas mean bone purchase deviations were significantly lower for experienced surgeons in the thoracic region (*p* = 0.002), but differences were not significant in the lumbar and sacral regions. The mean time taken to drill the hole was significantly longer for the inexperienced surgeon (*p* < 0.001). PC-PSRF using EOFG was successfully demonstrated at T13-L1 and L7-S1.

**Conclusion:**

The technique was feasible and allowed for percutaneous fixation of the thoracolumbar and lumbosacral segments. Previous experience in open instrumented spinal surgery is beneficial for safe and accurate application.

**Clinical significance:**

The PC-PSRF technique using EOFG guidance warrants future clinical studies to investigation a potential role in reducing pain, hospitalisation length, muscle injury and need for surgical revision.

## 1 Introduction

There has been a recent increase in publications concerning minimally invasive surgery (MIS) techniques in veterinary orthopaedics, such as minimally invasive plate osteosynthesis (MIPO) and tarsal arthrodesis (MITA) (1). In veterinary spinal surgery, MIS has been described for percutaneous decompression of extruded or protrusive intervertebral discs (2). Percutaneous lateral vertebral body fracture stabilisation using external skeletal fixators has also been described (3). MIS techniques are expected to have multiple advantages over open techniques, such as reduced muscle trauma, preservation of muscle blood supply, reduced skin tension, improved patient comfort with reduced reliance on analgesia and reduced hospitalisation periods (1–3).

Vertebral fixation using polyaxial “pedicle” screws and interconnecting rods, have been described for several applications in dogs including lumbosacral degenerative stenosis (4) and cervical spondylomyelopathy (5). In man, pedicle screw fixation utilising a minimally invasive percutaneous technique was described over 20 years ago, a technique that relies upon manipulation of the screws from the skin surface, and placement of rod through the muscle using a specific insertion device (6). Subsequently, cannulated pedicle screws were developed that can be inserted percutaneously over a guide wire; a safe technique described for a range of spinal pathologies (7). In veterinary medicine, the placement of polyaxial pedicle screws (without percutaneous rod passage and screw manipulation) has been described in a cadaveric model using neuronavigation (8). The safety and accuracy of polyaxial cannulated vertebral pedicle or body screw and rod fixation (PC-PSRF) with specific percutaneous instrumentation is yet to be described for dogs.

Accurate and safe implant corridors are crucial in spine fixation surgery to avoid neurovascular structures. We recently described the safety and accuracy of an ‘end-on’ fluoroscopic drill hole position technique (EOFG) in dog cadavers, that was superior to free hand placement (9). EOFG might be useful for initial guide-wire placement for PC-PSRF in dogs.

Our aim was to develop and describe a technique for PC-PSRF using EOFG guide wire placement in a dog cadaveric model, demonstrating its feasibility and reporting the accuracy and safety of the technique. We hypothesised PC-PSRF using EOFG would be a safe technique, irrespective of the experience of the surgeon, but that experienced surgeons are faster and more accurate than inexperienced surgeons. Given the EOFG technique was less accurate in the thoracic region (9), we also hypothesised this would be the case for PC-PSRF.

## 2 Materials and methods

### 2.1 Study design

This first part of this study was a cadaveric operator comparison study. The second part was a feasibility study to demonstrate the application of the spinal instrumentation in dogs. The study subjects were three dog cadavers weighing > 25 kg, euthanised for reasons unrelated to spinal disease, with no previous clinical history suggestive of spinal disease. Use of the cadavers for minimally invasive spinal surgery was approved by written owner consent following institutional ethical approval.

### 2.2 Subject preparation and technique planning

Cadavers were utilised for the study within seven days of euthanasia. A planning CT (Siemens Somatom Scope, 16 slice) was first performed with the cadaver in sternal recumbency. The helical scan protocol encompassed images from the first thoracic vertebra to the first coccygeal vertebra. Acquisition parameters included a slice thickness of 0.75 mm, a pitch of 1, a matrix size of 512×512 and utilisation of a bone kernel for image processing. In-silico planning for both techniques was performed in commercially available DICOM (digital imaging and communications in medicine) viewing software (OsiriX MD Dicom Viewer Pixmeo Sarl^®^, version 13.0.1, Geneva, Switzerland) with 3D multi-planar reconstruction (MPR) perpendicular to the long axis of the vertebral column. Region of interest (ROI) tools for measuring angle and distance were used (9).

The planning CT was first used to confirm that there were no incidental diseases affecting vertebral morphology (malformation, osteolytic or productive lesions related to suspected spinal neoplastic, inflammatory, or infectious disease). Plans were then produced for all 68 drilled holes, according to the EOFG technique (9). The ideal insertion points and angulations determined using three-dimensional reconstructions in ‘thick slab mode’ adjusting the mean slice thickness to between 15 and 20 mm (interval 0 mm), orientated to the angle of implant insertion, such that the reconstructed image in a dorsal oblique plane would mimic the desired appearance of intra-operative fluoroscopy.

### 2.3 Cadaveric technique

Cadavers were prepared by clipping the hair over the dorsum from the mid-thorax to the tail base. Holes were drilled by one of two experience levels: ‘experienced’ being board-certified neurologists with more than 15 years of experience of open instrumented spinal fixation surgery (CD or JR) versus ‘inexperienced’ being a third-year ECVN resident (FC) with 3 years of supervised residency training in spinal surgery but without independent experience performing spinal fixation procedures. The operated side was randomised by experience level. The time taken to drill each hole (from initial assessment of insertion position to penetration of the trans cortex) was recorded.

**Figure 1:**
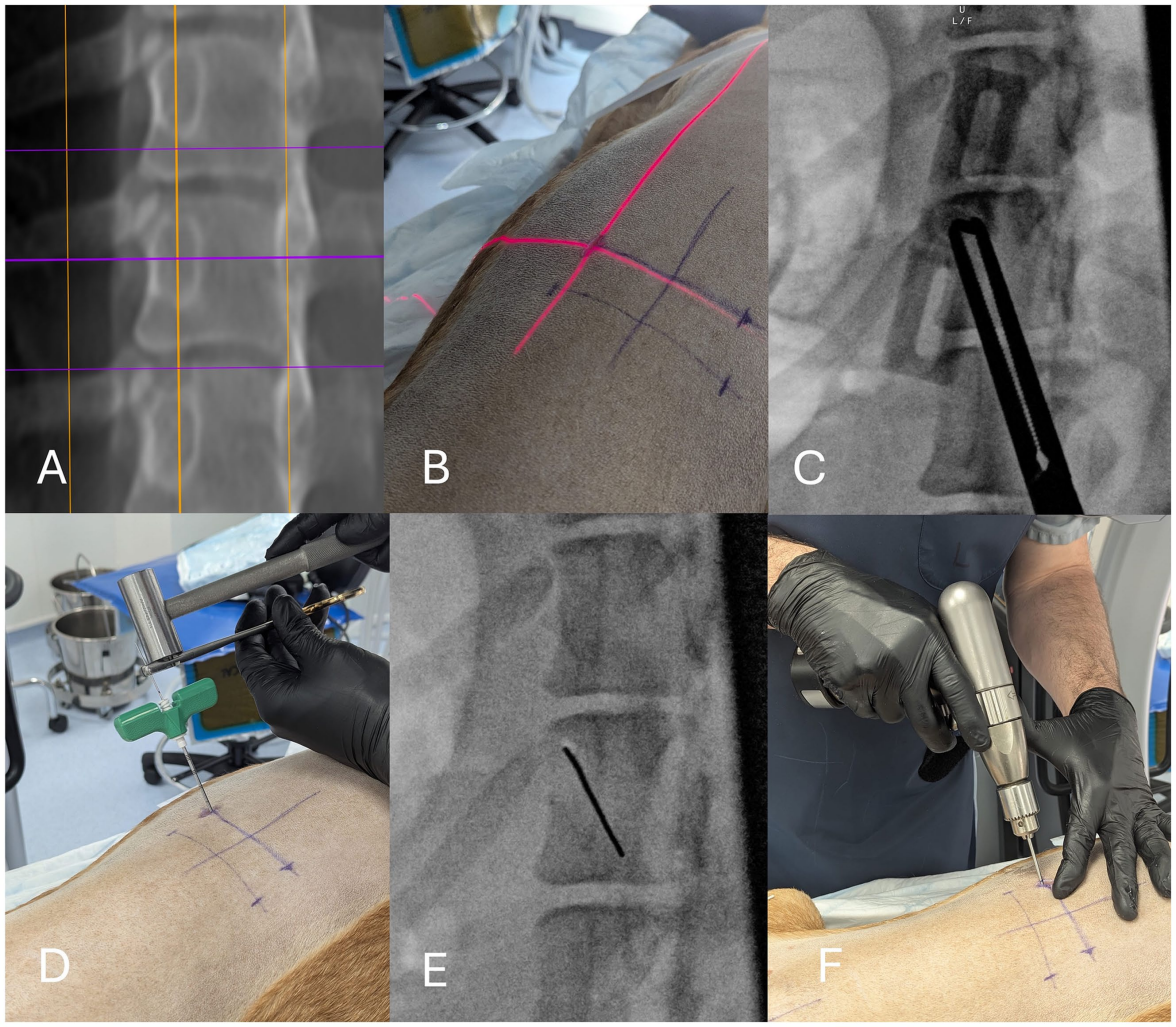
Safety and accuracy study, method of percutaneous fluoroscopic guided drill hole placement. The procedure is planned from CT, using a multi-planar reconstruction along the intended drill hole trajectory, with slice thickness of 15–20 mm **(A)**. The C-arm guidance laser and intermittent fluoroscopy exposures are used to determine the ideal location for the initial skin and fascial incision **(B)**. A Jamshidi needle is inserted to the level of the bone along the laser trajectory. Long forceps are used to help manipulate the needle such that the cross-sectional area of the needle is reduced to being as small as possible over the intended insertion point **(C)**. The handle is then struck gently with a mallet to penetrate the bone. The stylet is removed and a 1.1 mm k-wire is inserted into the needle lumen and into the cancellous bone. The k-wire is grasped by a pin-pulling forcep and the forcep is struck by a mallet to forcibly insert the k-wire towards the trans-cortex **(D)**. Fluoroscopy is used to check the position and angulation of the k-wire **(E)**. A cannulated drill bit is then used to drill a bi-cortical hole **(F)**.

Local radiation safety rules concerning fluoroscopy were obeyed, including the use of personal and finger ring dosimeters. C-arm (Fujifilm FDR Cross) angulation was set according to the in-silico plan and was orientated to the approximate insertion point on the vertebral body or pedicle using its guidance laser (Figure 1). X-ray projection parameters varied according to the physical properties of each cadaver (voltage 55–75 kV, collimation 14 cm, fluoroscopic mode at 25 frames per second). To limit radiation exposure, the continuous fluoroscopy function was limited to a fraction of a second with each exposure.

Dissimilar to the open EOFG technique (9), a 16-gauge Jamshidi needle was used to identify the ideal insertion point and penetrate the cis-cortex. Firstly, a 10 mm stab incision was made through the skin, subcutaneous fat and fascia using a no.11 scalpel blade. The Jamshidi needle was then advanced through the muscles to contact the surface of the vertebral lamina, pedicle or body. The Jamshidi needle was first held slightly oblique to the ideal insertion angle, using long-handled forceps (to prevent accidental exposure of the operator) to determine the tip of the needle overlay the ideal insertion point, using a fluoroscopic exposure. Then the body of the Jamshidi needle was re-orientated such that subsequent exposures demonstrated the smallest possible cross-sectional area of the needle. An orthopaedic mallet was then used to hammer the needle through the cis-cortex, with care taken to not cause excessive deviation of the intended insertion angle. The fluoroscopic exposure could be repeated if the surgeon wished to check the position of the needle. After this, the C-arm was moved parallel to the operating table to give the surgeon working space above the Jamshidi needle.

Following removal of the stylet, a 1.0 mm sharp k-wire was inserted into the lumen of the Jamshidi needle for introduction into the cancellous medullary bone. The k-wire was held and clamped by pin-pulling forceps, perpendicular to the k-wire, approximately 1 cm from the Jamshidi needle handle. The forceps were then gently hammered by the mallet, forcibly inserting the k-wire into the cancellous medullary bone. The Jamshidi needle was then gently removed from the bone, leaving the k-wire in position. A cannulated drill bit was then used to drill the bi-cortical hole, with drill depth assessed by tactile feedback from the trans cortex.

For the first part of the study (cadavers 1 and 2), a 3.5 mm diameter cannulated drill bit was used to mimic the smallest size of the cannulated polyaxial screws. For the second part of the study (cadaver 3), a 2.3 mm diameter cannulated drill bit was used, and the drill was only advanced to the trans-cortex. The only additional step was the insertion of a drill guide sleeve (specific to the percutaneous instrumentation) over the guiding k-wire.

### 2.4 Feasibility study

**Figure 2:**
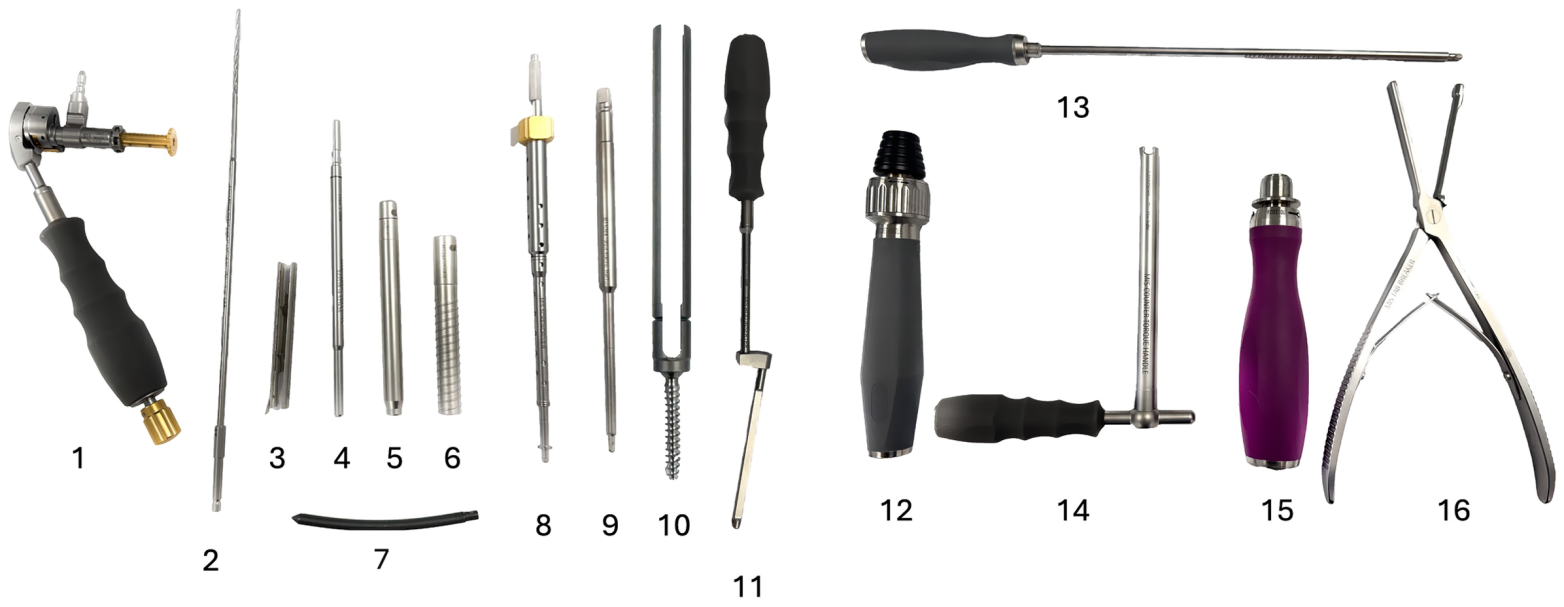
Aesculap® Ennovate® cervical minimally invasive instrumentation (not to scale). 1. Universal drill guide body; 2. Cannulated drill bit; 3. Extended tab sleeve for minimally invasive screws; 4. Drill guide body; 5. Dilator sleeve, small; 6. Dilator sleeve, large; 7. Curved rod; 8. Minimally invasive screw driver; 9. Cap tightener/remover; 10. Minimally invasive screw, 3.5 mm diameter; 11. Rod inserter; 12. Screw driver handle with ratchet; 13. Cap inserter; 14. Counter torque; 15. Torque limiting handle; 16. Tab breaker.

A commercially available system (Aesculap^®^, Ennovate^®^ Cervical) (Figure 2) was applied in cadaver 3 for fixation of T13-L1 and L7-S1. The system consists of 3.6, 4.0 and 4.5 mm diameter titanium polyaxial screws. They are cannulated, accepting a 1.0 mm (3.6 mm screw) or 1.5 mm (4.0 and 4.5 mm screws) guide wire, with a tapering shape for ease of insertion and increased strength at the neck of the screw, below the top loading inverted U shape head (tulip), which accepts a 4.0 mm titanium or cobalt chrome rod, and locking set screw. The minimally invasive version of the screws has extended tulip ‘petals’ or ‘tabs’, which can be manipulated/aligned external to the body, and a specialised rod insertion tool that allows the passage of a pre-curved rod with a blunt point, using the extended tabs for guidance between the tulips, which can subsequently be broken off leaving nothing above the muscle layers.

This system is intended for cervical and upper thoracic surgery in man (10), which was selected for this study because it offers suitable screws sizes for the canine spine. The manufacturer has a recommended methodology for implant insertion, including the option for neuronavigation. However, it was necessary to adapt a k-wire technique for use in dogs. This is due to more extreme variances in vertebral morphology and more contoured bony insertion points to access the vertebral pedicle or body, the latter of which is more often utilised for secure vertebral fixation in dogs. In addition, intra-operative cross-sectional imaging and neuronavigation is not routinely available for companion animals. 3.6 mm diameter screws and 60 mm length rods were selected because they would be suitable for vertebral fixation in a range of large-breed cadavers.

**Figure 3:**
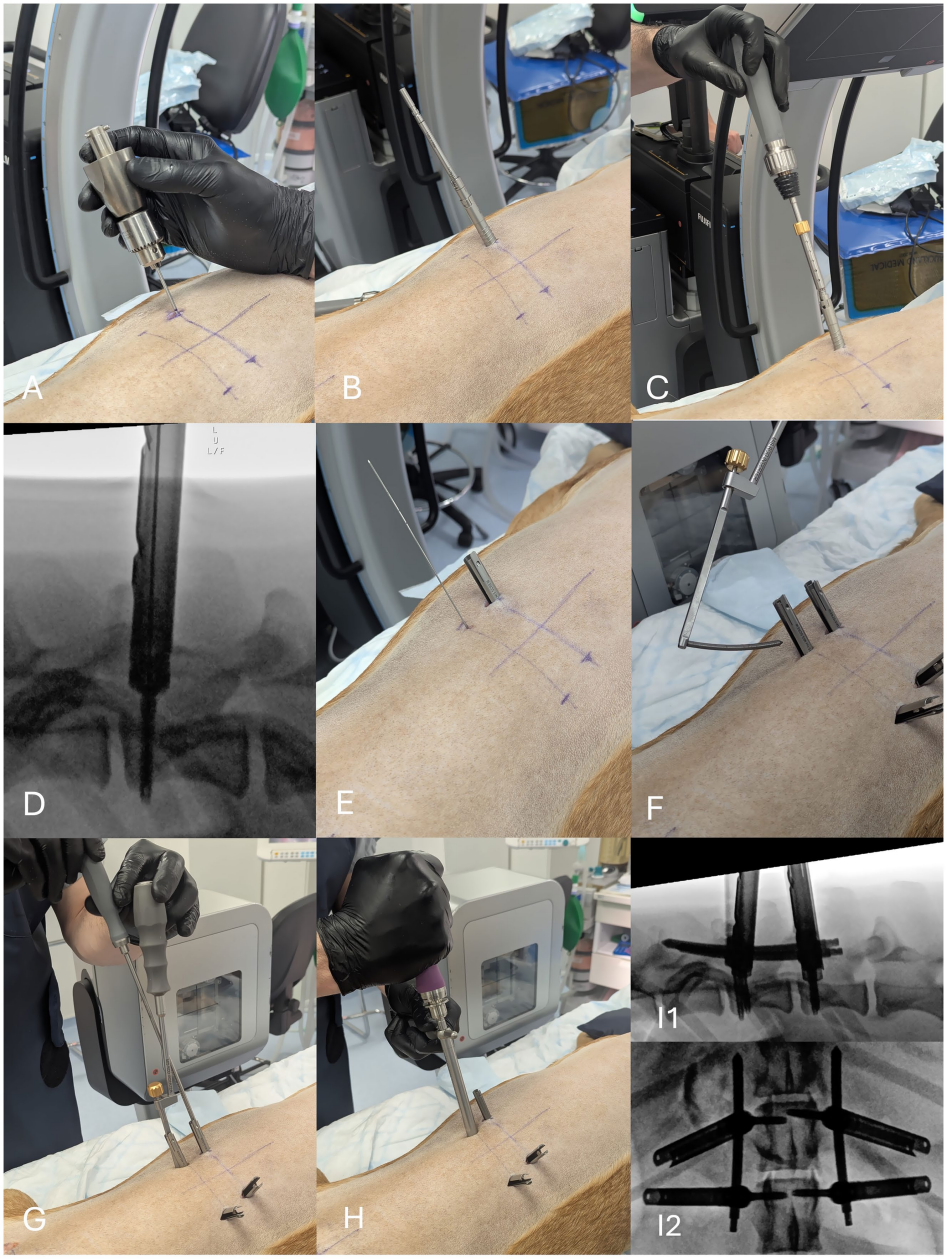
Feasibility study method utilising the Aesculap® Ennovate® cervical minimally invasive instrumentation in a dog cadaver at the level of T13-L1. After the k-wire guided drill hole is made (Figure 1), the drill bit is carefully removed from the k-wire **(A)** and sequential muscle dilators are used to dilate the muscles using a twisting motion **(B)**. The inner dilators were then removed, prior to the insertion of a 3.6 mm diameter cannulated screw with extended tab sleeve over the k-wire **(C)**. Under lateral fluoroscopic guidance, the screw was inserted to the desired depth **(D)**. The screw driver is detached from the screw and the k-wire process is repeated **(E)**. The extended tab sleeves were manipulated to facilitate ease of passage of the curved rod, which was inserted using the system specific rod inserting device with a curved motion **(F)**. With the rod held in position by the rod inserting device, the set screw inserter is used to insert and begin tightening of the set screw **(G)**. After release of the rod from the rod inserting device, the counter torque and torque limiting handle/driver are used to tighten the set screws to 2.8 Nm **(H)**. The extended tab sleeves were removed and the position of the construct was checked with fluoroscopy in lateral **(I1)** and dorsoventral **(I2)** planes.

Following completion of the drill hole (Figure 1), the cannulated drill bit was withdrawn without dislodging the k-wire, prior to the screw insertion process (Figure 3). A drill guide sleeve serves as the first stage of three serial dilators, to dilate the muscles away from the drill hole. After muscle dilation, the first and second stage dilators are removed, leaving the third as a channel for passage of the screw. Care was taken to not deviate excessively from the screw insertion angle, although the lumbodorsal fascia typically held the largest dilator in the correct orientation. The cannulated screw is then loaded onto the system specific screwdriver and is inserted in a bi-cortical fashion over the k-wire, with tactile feedback and lateral fluoroscopic exposure confirming engagement of the trans-cortex. Lastly, the k-wire is removed. After remaining screw insertion is completed, two 60 mm long pre-contoured rods were inserted through the muscle and into the tulips, in a sweeping motion from caudal to cranial, using the specific rod-inserting device. The extended tab sleeves help to guide the rods into position. A counter torque device was used to stabilise the tulips whilst the set screws are tightened with a set torque of 2.8 Nm, as recommended by the manufacturer.

The long tulip tabs were left exposed for post-procedure imaging, for ease of screw removal at the end of the study.

### 2.5 Image evaluation

When all holes were drilled the cadaver was transferred for repeat CT of the spine with the position, acquisition protocol and field of view to match the previous scans. CT images were evaluated by one of the authors (CD) with Osirix using 3D-MPR in a bone window using ROI tools. CT datasets were anonymised prior to analysis, and the evaluator was blinded to surgeon experience level and drilling side. Continuous data for drill-hole accuracy was determined from two factors as previously described (9); namely, ‘angle deviation’ (in degrees) from the planned angled trajectory of insertion, and ‘depth deviation’ (in mm) below the planned insertion depth was recorded (representing the difference between the achieved drilling depth and the planned insertion depth, used as a surrogate measure of adequacy of bone purchase). Where this latter value was negative (i.e., greater bone purchase achieved) it was recorded as zero. Safety was determined categorically using the modified Zdichavsky classification (11). The holes drilled as part of the feasibility study were included in the final analysis.

### 2.6 Statistic analysis

Data sets were analysed according to all data, data by surgeon (experienced versus inexperienced) and data by spinal region (thoracic, lumbar or sacral spine). All data sets were assessed for normal distribution. Statistical significance was set at *p* < 0.05.

In determining drill-hole safety, modified Zdichavsky grade 111a holes were categorised as “unsafe” and all other grades categorised as “safe”. Modified Zdichavsky grade 1 holes were categorised as “optimal” and all other grades categorised as “suboptimal”. Differences in safety category of the two methods were incorporated into multivariable logistic regression analysis to take cadaver number, surgeon experience, and spinal region into account.

In determining drill-hole accuracy, the differences in angle and depth deviations between the two levels of surgeon experience were compared in a paired vertebrae analysis at the univariable level using either paired t-tests (if normally distributed) or a Wilcoxon signed rank test (non-normally distributed) first considering all drill-holes and then by spinal region.

The differences in time taken between the two techniques was compared for paired vertebrae analysis using either paired t-tests (if normally distributed) or a Wilcoxon signed rank test (non-normally distributed).

## 3 Results

Cadavers used for the study were of the following breeds and body weights: Cadaver 1; Golden Retriever, 35 kg, Cadaver 2; Golden Retriever, 32 kg, Cadaver 3; Labrador, 28 kg. Mean age was 6 years (range: 2–12 years), and there were 2 male and 1 female dogs. None of the vertebral columns had radiographic evidence of bony changes.

**Figure 4:**
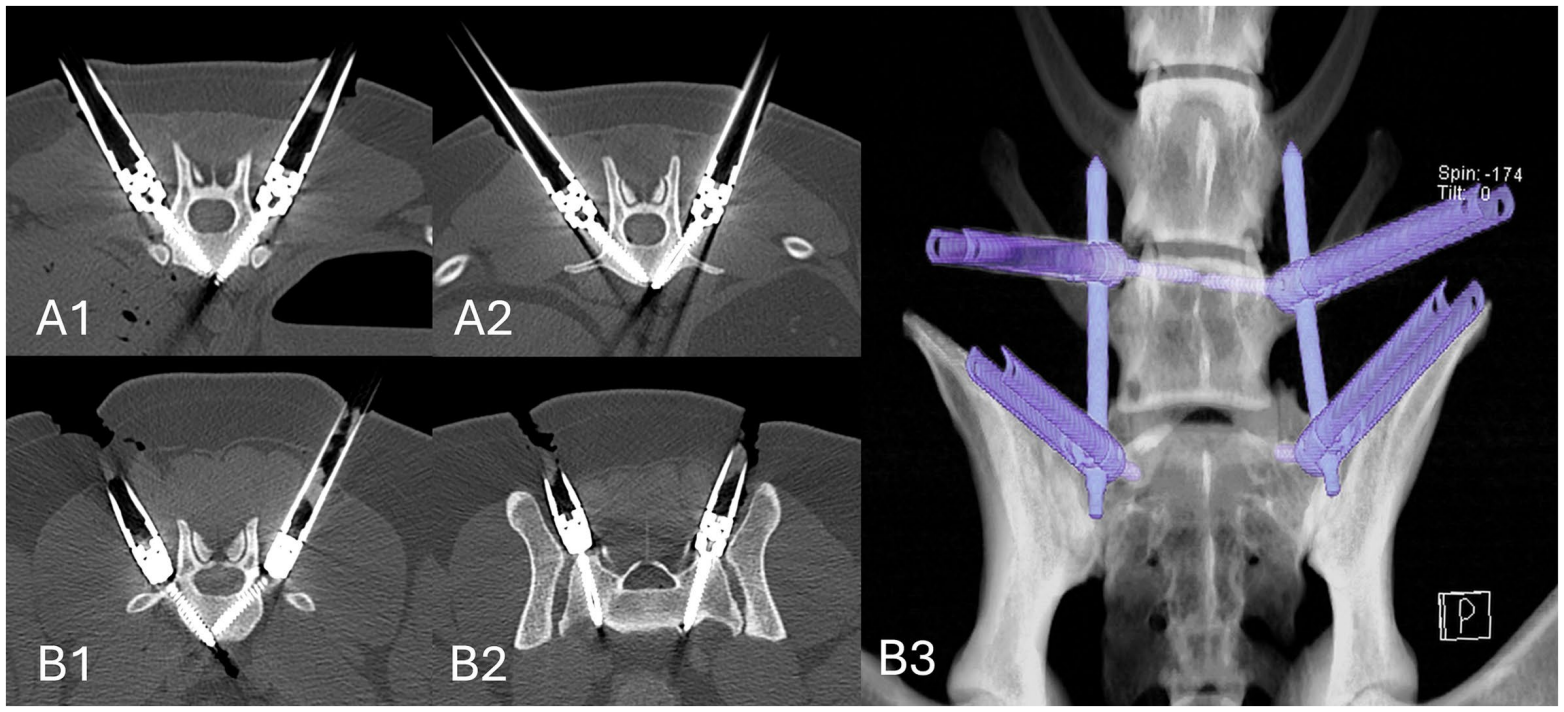
Feasibility study post-procedural CT scans in a bone window, reconstructed to demonstrate the insertion position and angles. Thoracolumbar fixation was performed with 3.5 mm screws, inserted into the vertebral bodies of T13 **(A1)** and L1 **(A2)**. Lumbosacral fixation was performed with 3.5 mm screws, inserted into the body of L7 **(B1)** and the alar wings of the sacrum **(B2)** in a converging pattern, which was considered necessary for the rods to be passed lateral to the facet joints **(B3)**.

Drill-hole safety data is summarised in Table 1. The experienced surgeons did not drill any unsafe holes (0/34), whereas the inexperienced surgeon drilled 4/34 (11.8%) unsafe holes. Type 2b errors were the most common error experienced (10/68, 14.7%) and were more common in the lumbar region (6/10 of the type 2b errors). Overall, drill hole placement for experienced surgeons was categorised as optimal in 28/34 (82.4%) of holes, in comparison to 14/34 (41.2%) by the inexperienced surgeon (*p* < 0.001). Percentages for optimal drill hole placement for experienced surgeons was higher in the thoracic region (84.6%) in comparison to the lumbar (66.7%) segments, whereas results were similar for the inexperienced surgeon between segments (30.8 and 31.3% respectively). In the multivariate analysis, optimal drill hole placement was significantly associated with surgeon experience (*p* < 0.001, OR = 9.66, 95% CI: 2.95–36.5) whereas cadaver number and spinal region did not reach significance (Figure 4).

**Table 1:** Summary of drill-hole safety data.

Region	Surgeon	Modified Zdichavsky grade					Optimal %
		1	2a	2b	3a	3b	
ALL	Experienced	28	2	4	0	0	82
	Inexperienced	14	3	6	4	7	41
Thoracic	Experienced	11	1	1	0	0	85
	Inexperienced	4	0	3	3	3	31
Lumbar	Experienced	11	2	3	0	0	69
	Inexperienced	5	3	3	1	4	31
Sacral	Experienced	5	0	0	0	0	100
	Inexperienced	5	0	0	0	0	100

Accuracy data is summarised in Table 2. Overall, there was a significant difference in mean (± SEM) angle deviation between experienced (4.80° ± 0.56°) and inexperienced (7.78° ± 1.21°) surgeons (*p* = 0.04). There was also a significant difference in mean bone purchase deviation between the experienced (1.59 mm ± 0.34 mm) and inexperienced (5.17 mm ± 1.16 mm) surgeons (*p* < 0.001). In the paired univariate analysis by region, mean angle deviations were not significantly different between experience levels by region, whereas mean bone purchase deviations were significantly lower for experienced surgeons (2.14 mm ± 0.63 mm) in comparison to inexperienced surgeons (7.72 mm ± 2.11 mm) in the thoracic region (*p* = 0.002), but differences were not significant in the lumbar (1.31 mm ± 0.50 vs. 4.28 mm ± 1.62, *p* = 0.17) and sacral (0.98 mm ± 0.42 vs. 1.38 ± 0.81 mm, *p* = 0.38) regions.

**Table 2:** Summary of drill-hole accuracy data including results of univariable analysis.

Variable	Region	Surgeon		*p*-value
		Experienced	Inexperienced	
Angle deviation^a^	ALL	4.80 (0.56)	7.78 (1.21)	0.04*
Purchase depth deviation^b^	ALL	1.59 (0.35)	5.17 (1.56)	<0.001*
Angle deviation^a^	Thoracic	5.13 (0.72)	6.39 (1.49)	0.50
	Lumbar	5.62 (0.87)	7.86 (1.98)	0.31
	Sacral	1.33 (0.73)	11.13 (3.63)	0.06
Purchase depth deviation^b^	Thoracic	2.14 (0.63)	7.72 (2.11)	0.002*
	Lumbar	1.33 (0.50)	4.28 (1.62)	0.17
	Sacral	0.98 (0.42)	1.38 (0.81)	0.38

a Angle deviation in degrees (mean ± standard error of mean).

b Purchase depth variation in mm (mean ± standard error of mean).

*Denotes statistical significance (*p* value set at <0.05).

There was a significant difference (*p* < 0.001) in mean (± SEM) time taken to drill the hole between experienced (2 min 47 s ± 10 s) and inexperienced (5 min 21 s ± 28 s) surgeons.

**Figure 5:**
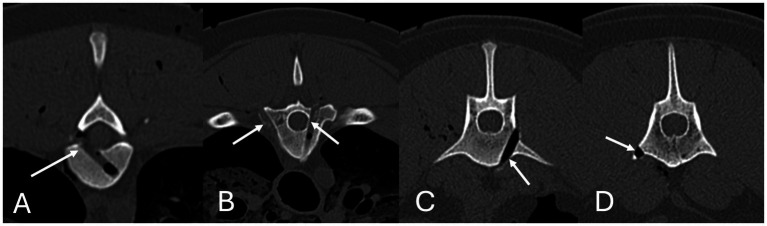
Safety and accuracy study, determination of drill hole errors according to the modified Zdichavsky grade, assessed using post-procedure CT scans in Osirix using multi-planar reconstructions in a bone window. Errors are highlighted by arrows. Type 3a error in the thoracic **(A)** spine. Type 2a (right arrow in image) and type 3b (left arrow in image) errors in the thoracic spine **(B)**. Type 2b **(C)** and 3b **(D)** errors in the lumbar spine.

In the feasibility study, the instrumentation successfully facilitated the minimally invasive, percutaneous placement of bi-cortical vertebral body screws with interconnecting rods at T13-L1 and L7-S1. Post-procedural CT confirmed accurate implant placement (Figure 5). At L7-S1, the screws were placed from parasagittal to converge towards the ventral mid-line such that the rods could be passed through the muscles lateral to the articular facet joints, without impediment from those joints.

## 4 Discussion

The EOFG technique was firstly described as method to predict implant position, relative to the vertebral canal, serving as an alternative method to post-operative CT (12). EOFG drill-hole placement was safe and accurate in comparison to freehand placement (9). In this study, we successfully developed and described a technique for PC-PSRF using EOFG with commonly accepted vertebral implant trajectories (13). We hypothesised that PC-PSRF using EOFG would be safe, irrespective of the experience of the surgeon, but that experienced surgeons are faster and more accurate than inexperienced surgeons. We could not fully accept this hypothesis, as whilst experienced surgeons achieved superior accuracy without unsafe drill holes, there were four unsafe holes drilled by the inexperienced surgeon that might have inadvertently led to spinal cord injury. Our results suggest PC-PSRF using EOFG may be best reserved for surgeons with significant experience of open instrumented spinal surgery.

Given EOFG was less accurate in the thoracic region (9), we also hypothesised this would be the case for PC-PSRF. However, PC-PSRF was associated with the least optimal results in the lumbar region. One possible explanation for this finding is that the Jamshidi needle may be more prone to slipping ventrally on more obliquely contoured bone at the point where implant insertion into the vertebral body is preferred (13). In the thoracic region, the rib head and lamina overlying the pedicle is flatter. Our results compare favourably to percutaneous screw placement using conventional fluoroscopy techniques in man, where according to the original Zdichavsky classification safety scheme (14), 98% of screws were determined to have a good or excellent position (15). However, our study concerned far fewer screw holes and further study, in a more diverse population (dogs of different breeds and body sizres), is required to assess the safety and accuracy of the technique in a clinical setting.

MIS of the spine is not commonly in veterinary medicine; however, there has been an increase in popularity due to numerous potential advantages over open techniques (1–3). In the human literature, MIS techniques are reported to reduce the length of hospitalisation, blood loss, reduce muscle trauma, skin tension and improve patient comfort (16). Percutaneous screw instrumentation has been described to enhance spinal stability and to promote fusion in patients with trauma, tumours, deformities, or degenerative pathology (16). While open approaches to fusion involve extensive muscle stripping, percutaneous techniques utilize intraoperative imaging to guide pedicle screw placement through a small incision with blunt, muscle splitting dissection, thereby decreasing soft tissue trauma (17). Given that the paraspinal musculature of a dog provides dynamic stabilization to the vertebral column, counteracting sagittal flexion during different phases of gait and stance (18, 19), its preservation during surgery is likely to be of long-term functional benefit following surgery.

Fluoroscopic-assisted spinal stabilisation using spinal arch external skeletal fixators (20), unilateral uniplanar (3) and unilateral biplanar external skeletal fixators (21) has been described for dogs with vertebral fracture-luxations, with good clinical outcomes. These techniques rely on the lateral placement of vertebral body pins and their subsequent removal. Complications can arise from the persistence of percutaneous pins including pin tract exudation or infection and pin loosening (3). PC-PSRF has the advantage of allowing normal wound closure. Despite first being described over 20 years ago in human healthcare (6), PC-PSRF has not been described for use in companion animals. We demonstrated PC-PSRF to be feasible at both the thoracolumbar and lumbosacral regions. PC-PSRF has multiple potential indications for use in small animal spinal disorders, including trauma (3), infection (discospondylitis) (22) and degenerative spinal diseases such as chronic thoracolumbar intervertebral disc protrusions (23) and degenerative lumbosacral stenosis (DLSS) (24, 25). In DLSS, the volume of the intervertebral foramen and vertebral canal can be enlarged with the lumbosacrum in a flexed position (24) and therefore indirect neural decompression can possibly be achieved through vertebral stabilisation without an open approach to the vertebrae for laminectomy and/or foraminotomy (25). Because the vertebral alignment and angulation can change with positioning, obtaining a pre-operative CT image in a flexed position could improve surgical planning and help preventing unexpected intraoperative findings.

Fluoroscopy is associated with additional radiation exposure to both surgeon and patient. In the current study exposure times were kept to a minimum, but as time for screw hole placement was associated with surgeon experience, less experienced surgeons may require additional radiation exposure to achieve surgical objectives. No safety issues were identified during routine dose assessments following completion of the study. Very limited data has been reported in literature to help quantify this exposure and further research is needed to quantify radiation exposure for both patients and surgeons using this technique. An alternative radiation-safe option for guiding percutaneous pedicle screw placement is neuronavigation, which is performed following pre-operative cross-sectional imaging. In humans, navigation exhibits a higher accuracy and increased safety in pedicle screw placement than free-hand technique and use of fluoroscopy (26). The technique has been described for percutaneous placement of pedicle screws in dog cadavers (8), albeit without specific PC-PSRF instrumentation.

Limitations of this study include the small sample size and the ex-vivo study design, which as previously mentioned may not fully represent the accuracy and safety in a clinical setting. This study was performed exclusively in large breed cadavers with normal vertebral column anatomy, and instrumentation was limited to two consecutive vertebrae within the thoracolumbar and lumbosacral regions. Therefore, the results may not be directly applicable to dogs of different breeds or body sizes, to vertebral columns with pathological changes, or to cases requiring longer constructs. Additionally, more complex clinical conditions, such as spinal fracture-luxation or cases requiring vertebral reduction or removal of unstable bone fragments, may be better managed with an open technique. Because multiple drillings were performed within the same cadavers, some measurements may not represent fully independent observations and may partly reflect specimen-specific anatomical characteristics. As PC-PSRF required surgical experience, there may be a steep learning curve to the technique. Given this, and the potential lack of availability of specialised operating room equipment and surgical instrumentation, there may be limited scope for widespread uptake of the procedure.

## 5 Conclusion

This ex-vivo experimental study demonstrates a feasible technique for percutaneous pedicle screw and rod fixation of the thoracolumbar and lumbosacral spine, under fluoroscopic guidance. Although prior experience in open instrumented spinal surgery is essential for its effective application, the technique appears a safe therapeutic option. Further *in vivo* studies are required to validate its clinical safety, accuracy and efficacy, and to establish its potential role in reducing hospitalisation time, tissue trauma, neurovascular injury, and the need for revision surgery.

## Data Availability

The original contributions presented in the study are included in the article/supplementary material, further inquiries can be directed to the corresponding author.
